# Correction: Akagi et al. Micromagnetic Study of the Dependence of Output Voltages and Magnetization Behaviors on Damping Constant, Frequency, and Wire Length for a Gigahertz Spin Rotation Sensor. *Sensors* 2023, *23*, 2786

**DOI:** 10.3390/s23156748

**Published:** 2023-07-28

**Authors:** Fumiko Akagi, Terumi Kaneko, Hirotada Kan, Yoshinobu Honkura, Shinpei Honkura

**Affiliations:** 1 Department of Applied Physics, School of Advanced Engineering, Kogakuin University, Tokyo 163-8677, Japan; s419023@g.kogakuin.jp; 2 Graduate School of Electrical Engineering and Electronic, Kogakuin University, Tokyo 163-8677, Japan; cm21011@g.kogakuin.jp; 3 Magnedesign Co., Ltd., Nagoya 470-2414, Japan; yoshinobu.honkura@magnedesign.co.jp (Y.H.); shinpei.honkura@magnedesign.co.jp (S.H.)

The authors wish to make the following corrections to the original paper [1].

## Text Correction

There were two errors in the original publication in Section 5.3. “Relationship between External Magnetic Field and Output Voltage for Each Axial Length”.

The sentence “Figure 12 shows the experimental time variation of magnetization in the *z*-axis at the maximum output voltage for each axis length.” should be changed to “Figure 12 shows the measured values of the relationship between the external magnetic field and the output voltage.”.

The sentence “These trends of the experiments also coincide with the simulation results shown in Figure 11.” should be changed to “These trends of the experiments also coincide with the simulation results shown in Figure 10.”.

## Error in Figure

In Section 5.3. “Relationship between External Magnetic Field and Output Voltage for Each Axial Length”, there were errors in the description notations of the graph lines in Figure 12. The correct figure is shown below.


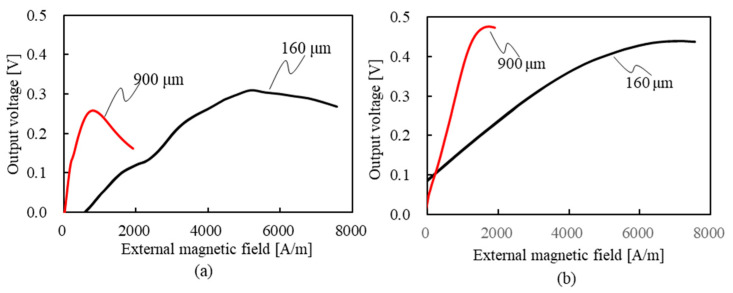


The authors state that the scientific conclusions are unaffected. This correction was approved by the Academic Editor. The original publication has also been updated.
